# Schwann-like cells seeded in acellular nerve grafts improve nerve regeneration

**DOI:** 10.1186/1471-2474-15-165

**Published:** 2014-05-21

**Authors:** Lihong Fan, Zefeng Yu, Jia Li, Xiaoqian Dang, Kunzheng Wang

**Affiliations:** 1 The first department of Orthopedics, the Second Affilliated Hospital of Xi’an Jiaotong University, No. 157 Xiwu Road, Xi’an, Shaanxi Province 710004, China

**Keywords:** Acellular nerve grafts, Schwann-like cells, Bone marrow-derived mesenchymal stem cells, Nerve repair

## Abstract

**Background:**

This study evaluated whether Schwann-like cells (SLCs) induced from bone marrow-derived mesenchymal stem cells (BM-MSCs) transplanted into acellular nerve grafts (ANGs) could repair nerve defects compared with nerve isografts and ANGs with BM-MSCs.

**Methods:**

BM-MSCs extracted, separated and purified from the bone marrow of rats, and some of the BM-MSCs were cultured with mixed induction agents that could induce BM-MSCs into SLCs. Either SLCs or BM-MSCs were seeded onto 10-mm ANGs, and the isografts were chosen as the control. The walking-track test, tibialis anterior muscle weight measurement, electrophysiological examination, toluidine blue staining, transmission electron micrographs and immunostaining of S-100 and VEGF in these three groups were evaluated in a 10-mm rat sciatic injury-repair model.

**Results:**

The walking-track test, tibialis anterior muscle weight measurement and electrophysiological examination of the sciatic nerve suggested the groups of ANGs with SLCs and isografts obtained better results than the BM-MSC group (*P* < 0.05). Meanwhile, the results of the SLCs and isograft groups were similar (*P* > 0.05). All the histomorphometric analyses (toluidine blue staining, transmission electron micrographs and immunostaining of S-100 and VEGF) showed that there were more regenerating nerve fibers in the group of ANGs with SLCs than the BM-MSCs (*P* < 0.05), but there was no significant difference between the SLC and isograft groups (*P* > 0.05).

**Conclusions:**

SLCs seeded in ANGs and isografts show better functional regeneration compared with BM-MSCs seeded in ANGs. Additionally, SLCs combined with ANGs present almost the same outcome as the isografts. Therefore, SLCs with ANGs can be a good choice in nerve defect repairs.

## Background

Trauma-induced peripheral nerve injuries affect approximately 2.8% of patients who undergo surgical intervention [1]. Unfortunately, in many cases, patients who suffer peripheral nerve injuries experience a permanently impaired quality of life.

To solve this medical issue, a range of studies and clinical trials have been performed to repair the nerve injuries. Among all the approaches to repair nerve injuries, autologous nerve bridges, also known as isografts, which are regarded as the clinical “gold standard” for repairing nerve gaps, are the most effective method; however, this method is also restricted due to some problems, especially limited donor nerve resources [2]. Therefore, to address this issue, numerous synthetic and natural biomaterials have become alternatives. Earlier studies demonstrated some alternatives to induce axon regeneration across nerve gaps, but their effectiveness was limited [3,4]. The internal structure and extracellular matrix (ECM) components of nerve grafts have been demonstrated to be an ideal candidate for guiding cell migration and nerve fiber elongation [5,6]. Acellular nerve grafts derived from peripheral nerves maintain the original structures and ECM components and cause a low host immune response, even though the grafts are taken from xenogeneic animals [7].

Schwann cells (SCs) play an important role in nerve generation, such as producing neurotrophic factors and causing neurite outgrowth from neurons [8]. Therefore, SC treatment is a good option for nerve injury. Some groups have supplemented acellular nerve grafts with cultured SCs [9,10]. However, the application of SCs to treat nerve injures is rare, even obsolete, due to the disadvantages, such as limited resources, difficult purification protocol, and strong immune reactions [11,12]. Bone marrow-derived mesenchymal stem cells (BM-MSCs), a type of stromal cell present in the bone marrow, that possess multi-directional differentiation potential, have many clinical advantages, such as easy accessibility, rapid proliferation in culture and successful integration into the host tissue with a low immune response. Thus, many studies have been performed to determine whether BM-MSC therapy, as an alternative to SC therapy, is a promising approach for repairing nerve injuries [13,14]. Our earlier research and other studies suggest that SC transplantation induces better axon regeneration than BM-MSCs following peripheral nerve injury [15,16]. Luckily, some previous studies showed that BM-MSCs have the capability to differentiate into Schwann-like cells (SLCs) under specific conditions in vitro and that SLCs have some of the characteristics of SCs that are not present in BM-MSCs [17,18]. Furthermore, Walsh et al. [19] and Wang et al. [20] demonstrated that SLCs derived from precursor cells and different stem cells, respectively, help promote peripheral nerve regeneration.

Based on past studies, we hypothesize that the Schwann-like cells induced from bone marrow-derived mesenchymal stem cells could improve the extent of axon regeneration. The current study was designed to determine whether the addition of SLCs could increase axon regeneration through ANGs in a 10-mm rat sciatic nerve injury model.

## Methods

### Animals

Sprague–Dawley (SD) rats (180–220 g) and York pigs (approximately 200 kg) that were obtained from the Laboratory Animal Research Center of Xi'an Jiaotong University were used. All experimental animals were housed in a temperature- and humidity-controlled room with 12-h light/12-h dark and allowed free access to standard chow and water, and the experimental protocol was approved by the Animal Ethical Committee of Xi'an Jiaotong University. All surgical procedures were performed under general anaesthesia via the intraperitoneal injection of pentobarbital sodium.

### Preparation of acellular nerve grafts

Under aseptic conditions, the bilateral intercostal nerves of anesthetized York pigs were exposed, and the nerve segments were excised to prepare the ANGs. The connective tissue of the nerves was removed under a light microscope, and the nerves were then immediately placed in sterile distilled water and cut into 10-mm segments. Acellular nerves were prepared via optimized chemical processing, and all the subsequent steps were conducted based on the previously developed protocol [21]. Briefly, the nerve tissues were immersed in 0.25% trypsin for 30 min at 37°C, and then rinsed in deionized distilled water for 4 h. Subsequently, the water was aspirated and replaced with a solution containing 125 mM SB-10, 10 mM phosphate, and 50 mM sodium. The nerves were agitated for 15 h. The tissue was rinsed once for 15 minutes in a washing solution of 50 mM phosphate and 100 mM sodium. Next, the washing solution was replaced with a solution containing 0.14% Triton X-200, 0.6 mM SB-16, 10 mM phosphate, and 50 mM sodium. After agitating for 24 h, the tissue was rinsed in a washing solution 3 times at 5 minutes per rinse. The nerve segments were again agitated in the SB-10 solution (7 h), washed once, and agitated in the SB-16/Triton X-200 solution (15 h). Finally, the tissue segments were washed 3 times for 15 minutes in a solution containing 10 mM phosphate and 50 mM sodium and then stored in the same solution at 4°C.

### Primary culture of Schwann cells

Primary Schwann cells were obtained according to the previously published protocols described by Kaewkhaw et al. [22]. Briefly, the protocol was based on Schwann cell culture medium containing both mitogens (forskolin and N2 supplement plus bovine pituitary extract), to stimulate the growth of Schwann cells and the addition of D-valine to simultaneously restrict fibroblast overgrowth in Dulbecco’s Modified Eagle’s Medium (DMEM).

### BM-MSC preparation

Six to eight-week-old SD rats (n = 5) were euthanized using diethyl ether, and the long bones were collected under sterile conditions. The ends of each bone were then cut off. The bone marrow from each bone was collected by flushing the bone with α Minimum Essential Medium Eagle (αMEM) (Sigma, USA) containing 1000 U/ml Penicillin G (Sigma, USA). After filtering, the cells were centrifuged at 1000 × g for 5 min. The purified cells were finally dispersed in αMEM with 15% foetal bovine serum (Sigma, USA) containing 100 U/ml penicillin and 100 μg/ml streptomycin (Sigma, USA) [23].

### Preparation of SLCs

After being subcultured at a concentration of 1 × 10^6^ cells/cm^2^, BM-MSCs were incubated in αMEM containing 1 mM BME without serum for 24 h. The culture medium was then replaced with αMEM containing 10% FBS and 35 ng/ml at-RA (Sigma, USA). After three days, the cells were finally transferred to inducer medium containing αMEM, 10% FBS and trophic factors of 5 μM FSK (Calbiochem, CA), 10 ng/ml bFGF (Peprotech, UK), 5 ng/ml PDGF (Peprotech, UK), and 200 ng/ml HRG (R&D Systems, USA). The cells were cultured for 10 days [24,25].

### Immunostaining of cultured cells

Some of the SLCs cultured on chamber slides were fixed in 4% (w/v) paraformaldehyde at 4°C for 20 min. The cells were then incubated overnight at 4°C with primary antibodies against S-100 (rabbit polyclonal; 1:200; Santa Cruz, USA). The following day, the slides were incubated for 2 h with the secondary antibody (goat anti-rabbit; 1:100; Sigma, USA). The slides were examined under a microscope (Olympus SZ51) and the numbers of immuno-positive cells were counted in a minimum total of 100 cells per experiment. Cultures of Schwann cells and BM-MSCs were similarly stained as positive and negative controls according to the antibodies used.

### Construction of two types of nerve grafts in vitro

The isolated BM-MSCs and SLCs were utilized for seeding acellular-allogenic grafts. A single cell suspension containing 2% gelatine was prepared at a concentration of 2 × 10^7^ cells/ml, and the acellular nerves were pre-incubated in complete medium at 37°C for 3 h. A total volume of 30 μl of cell suspension was injected into the nerve graft using a microinjector, resulting in a loading of 6 × 10^5^ cells per graft. To perform the injection, under a dissecting microscope, the microinjector was inserted through the full length of the nerve segment, and the cells were injected in equal volumes at four evenly spaced points as the injector was withdrawn from the nerve. The nerve grafts implanted with BM-MSCs and SLCs were then immersed in DMEM supplemented with 10% FBS and incubated at 37°C with 5% CO_2_ under fully humidified conditions. After 3 days in culture, some nerve scaffolds containing BM-MSCs or SLCs were used to bridge the 10-mm nerve gap in vivo [26,27].

### Experimental design and surgery

All the SD rats were randomly divided into three groups (n = 15 per group): group A, SLC-laden ANG; group B, BM-MSC-laden ANG; and group C, isograft group. The rats were anesthetized with sodium pentobarbital, and the surgical site was shaved and sterilized with 75% ethanol 3 times. A skin incision was then made along the femoral axis, and the thigh muscles were separated, exposing the sciatic nerve. The right sciatic nerves were cut near the obturator tendon at mid-thigh and allowed to retract. The distal nerve stumps were transected to create 10-mm nerve defects. Both stumps of the sciatic nerve gap were bridged using SLC-laden ANG (ANG-SLCs) or BM-MSC-laden ANG (ANG-BM-MSCs) with 10–0 nylon monofilament. The transplantation of the isografts served as a control. For the isograft group, the 10-mm segments of sciatic nerves were resected, reversed 180°, and then sutured back onto the nerves. The wounds of the rats were rinsed with 40,000 U of gentamycin sulphate to reduce the chance of infection. The muscles were reapposed with 4–0 vicryl sutures, and the skin incision was clamped shut with wound clips. After surgery, the rats were placed under a warming light, allowed to recover from anaesthesia, and then housed separately with food and water ad libitum in a colony room maintained at a constant temperature (19 ~ 22°C) and humidity (40% ~ 50%) on a 12:12 h light/dark cycle.

### Walking-track test

The rat sciatic function index (SFI) score was evaluated as previously described [28-30]. At different time points (2, 4, 8, and 12 weeks), the hind feet of the rats were dipped in ink, and the rats were allowed to walk across a plastic tunnel so that the footprints could be recorded on paper loaded onto the bottom of the tunnel. The distance between the top of the third toe and the most posterior part of the foot in contact with the ground (print length, PL), the distance between the first and fifth toes (toe spread, TS), and the distance between the second and fourth toes (ITS) were measured on the experimental side (EPL, ETS, and EITS, respectively) and on the contralateral normal side (NPL, NTS, and NITS, respectively). The SFI was generated as follows: SFI = 109.5 × (ETS-NTS)/NTS-38.3 × (EPL-NPL)/NPL + 13.3 × (EITS-NITS)/NITS-8.8. In general, a SFI value approximately 0 indicated normal nerve function and a value of approximately −100 indicated total dysfunction.

### Electrophysiology

Electrophysiological tests were performed 12 weeks after the surgery. Under sodium pentobarbital anaesthesia, the previous surgical site at the mid-thigh level was re-opened, and the left sciatic nerve was re-exposed. Bipolar hooked platinum recording and stimulating electrodes were used to induce and record electrical activity. Bipolar stimulating electrodes were placed on the nerve proximal to the graft while recordings were obtained distal to the graft from the nerve and from the belly of the gastrocnemius muscle. The distance between the two electrodes was measured with a sliding calliper with 0.2 mm precision. Electrical stimuli were applied with an intensity of 1–30 mA to ensure the maximum waveform and prevent independent muscle contraction and also applied sequentially to the sciatic nerve trunk at the proximal and distal ends of the graft. The recordings of compound muscle action potential amplitude and nerve conduction velocity were recorded following a supramaximal stimulus delivered by the proximal electrodes. The procedures were performed on a heating blanket to maintain a body temperature of 37°C. The evoked peak amplitude of compound muscle action potential (CMAP) and the nerve conduction velocity in response to the stimuli were recorded using MedLab V6.0 system (Meiyikeji, China). The stimuli duration were 0.1-0.2 ms, and the stimulation frequency was 1 Hz.

### Tibialis anterior muscle weight measurement

12 weeks after surgery, the bilateral tibialis anterior muscle was dissected completely from the origin to the insertion and weighed immediately with an electron scale with 0.0001 g precision. The weight ratio of the operated side was expressed as a percentage of the muscle weight on the contralateral normal side.

### Histopathological observation

After being sacrificed, the never grafts of the rats were fixed by perfusion with 4% paraformaldehyde in 0.1 M phosphate buffer (pH 7.4), and the middle of the nerve grafts was harvested for TEM analysis. In addition, 500 nm semi-thin cross-sections of the specimens were cut and stained with 1% toluidine blue solution for the light microscopy study. In randomly selected high power fields, the total number of nerve fibers, myelin sheath thickness and myelinated fiber diameter of the regenerated nerves were analyzed with Image Pro Plus 6.0. The results were used to calculate the G-ratios (ratio of axon diameter to fiber diameter) [14,31].

### Immunostaining analysis of the regenerated tissues

Briefly, serial 8-μm-thick sections of the middle of the nerve graft were cut on a cryostat, mounted onto poly-L-lysine-coated slides, and subjected to immunohistochemical analysis with antibodies against S-100 (Sigma, 1:200 dilution) and VEGF (Sigma, 1:200 dilution). The immunoreactive signals were visualized by goat anti-mouse IgG (Jackson, 1:200 dilution). In randomly selected high power fields, the integrated optical density (IOD) of a positive immunological reaction was analyzed by Image-Pro Plus 6.0 [32,33].

### Statistical analysis

The data were expressed as the mean ± standard error of the mean (SEM). The analysis was performed using SPSS 13.0 software. One-way analysis of variance (ANOVA) followed by Dunnett’s test was used for the comparison among experimental groups. Non-parametric data were compared among the experimental groups using the Mann–Whitney U-test. *P* values less than 0.05 were considered statistically significant.

## Results

### Structure of the acellular nerves

**Figure 1:**
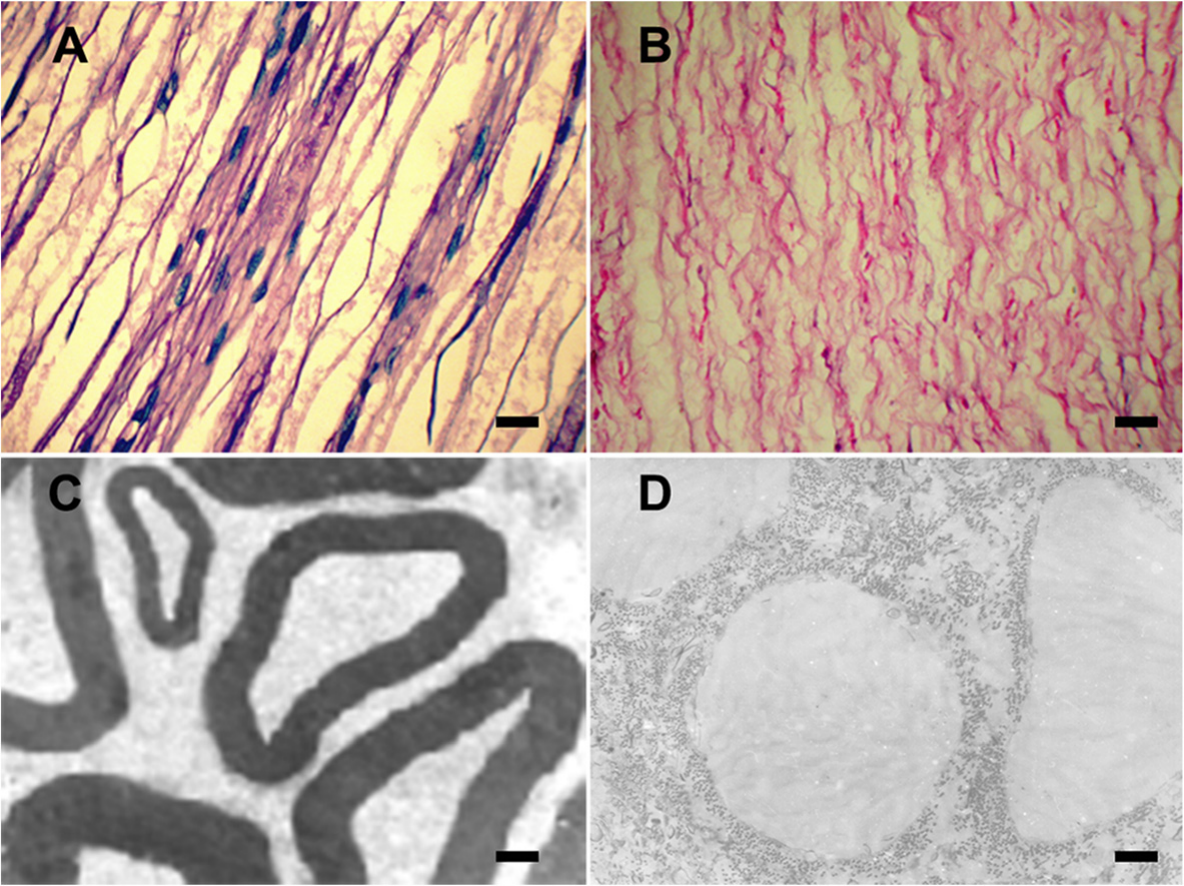
**H&E staining and transmission electron microscopy of normal nerves and acellular nerves.** H&E staining of the longitudinal sections of normal nerves **(A)** and acellular nerves **(B)**. Following decellularization, H&E staining showed that the endoneurial tubes were empty. Transmission electron microscopy of cross-sections of normal nerves **(C)** and acellular nerves **(D)**. TEM demonstrated that axons and myelin sheaths were absent in the acellular nerves. Scale bar = 20 μm **(A, B)**, 1 μm **(C, D)**.

For the acellular nerves, following decellularization, H&E staining showed that the endoneurial tubes were empty. The cells, axons and myelin sheath were removed completely, and the structural integrity of the basilar membrane was well retained (Figure 1B). In addition, the TEM ultrastructural analysis further demonstrated that the axons and myelin sheath were absent in the acellular nerves and that the collagen fibers were arranged regularly to construct the wall of the basal lamina tubes, which exhibited granular shapes (Figure 1D). Meanwhile, the normal nerve’s myelin sheath and cell nuclei were still intact (Figures 1A and C).

### Immunocytochemistry of SLCs

**Figure 2:**
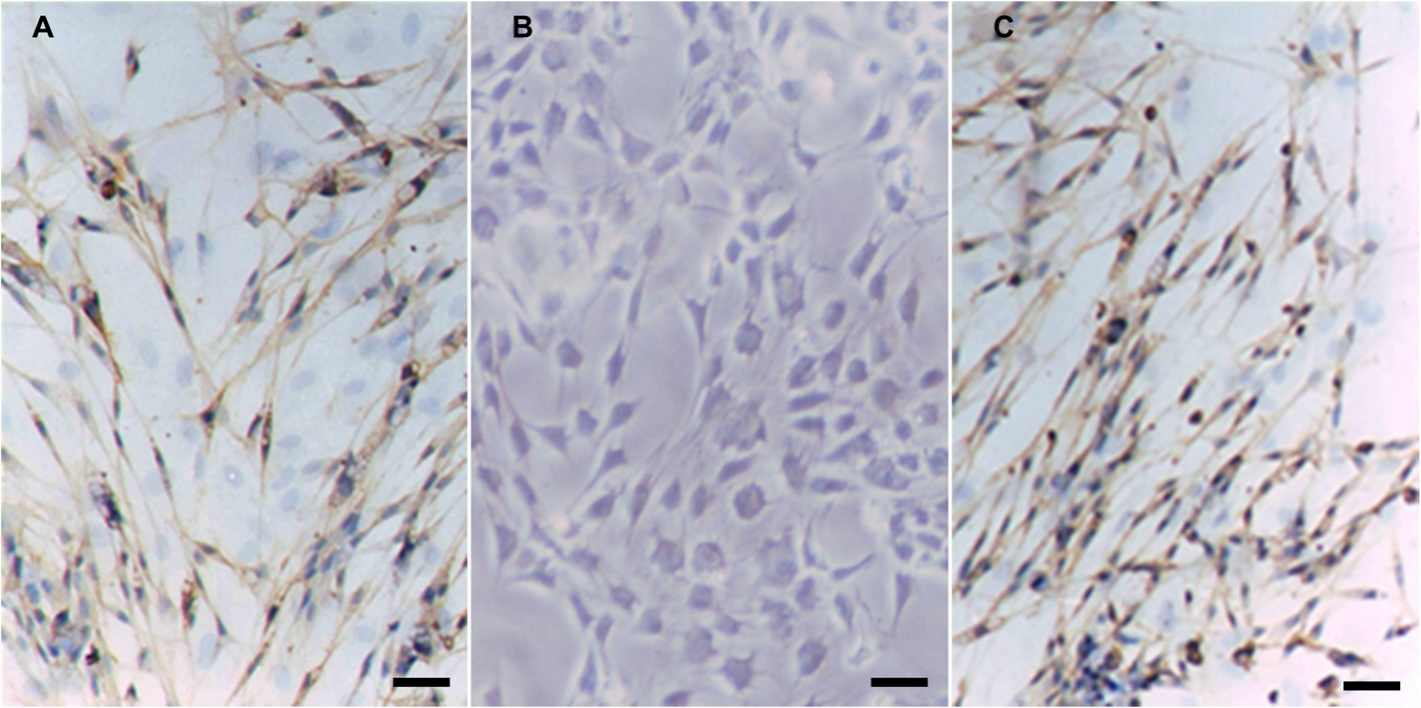
**Immunocytochemistry of S-100.** Immunocytochemistry of S-100 in SLCs **(A)**, BM-MSCs **(B)** and Schwann cells **(C)**. After induction, the SLCs became positive in response to the S-100 antibody (brown staining in the cytoplasm), whereas the BM-MSCs were negative. Schwann cells were used as a positive control. Scale bar = 20 μm.

We used the expression of S-100, which is a specific marker of Schwann cells, to estimate the characteristics of the SLCs. Almost all of the SLCs and SCs were positive in response to the S-100 antibody (Figure 2). After ten days of induction, the expression of the S-100 protein was demonstrated in the cytoplasm of the SLCs. In evaluating the differentiation of BM-MSCs to SLCs, immunocytochemical staining revealed that BM-MSCs to SLCs were positive for S-100 (82.3 ± 5.8%).

### Evaluation of the nerve regeneration and functional recovery

At no time point following surgery did the wounds of any study group show swelling and/or effusion. At 12 to 20 days post-operation, the wounds of all groups had healed spontaneously. Red swelling and trophic ulcers appeared on the footplates on the operated sides. Four to five weeks later, muscular atrophy of the extremities on the operated side occurred in all groups. After approximately 8 weeks, the footplate ulcers had almost healed, whereas the muscular atrophy and motor function were only partially recovered. There was minimal adherence between the transplants and surrounding tissues, and the morphology of the transplanted nerves was intact without signs of edema.

**Figure 3:**
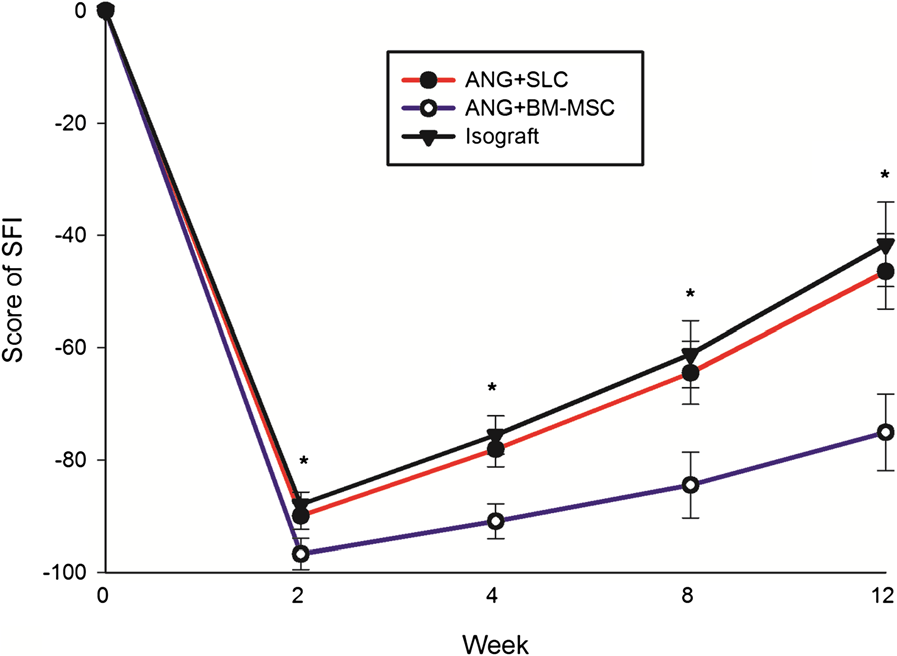
**The quantitative analysis of SFI value.** Typical walking tracks were obtained from the three experimental groups at 0, 2, 4, 8 and 12 weeks after surgery, and the SFI value was calculated. The SFI values in the isograft and ANG + SLC groups were significantly higher than in the ANG + BM-MSC group. The data are shown as the mean ± SEM, *P < 0.05 vs. the ANG + BM-MSC group (n = 15).

The efficacy of SLCs on motor functional improvement in the peripheral nerve-injured rats was evaluated based on SFI and electrophysiological analysis. The results showed that the SFI value was increased significantly in group A compared with group B at each time point tested (*P* < 0.05). No significant difference in the SFI value was observed between groups A and C (*P* > 0.05). These results indicated that the combination of SLCs was more effective in promoting the recovery of motor function than BM-MSCs (Figure 3).

**Figure 4:**
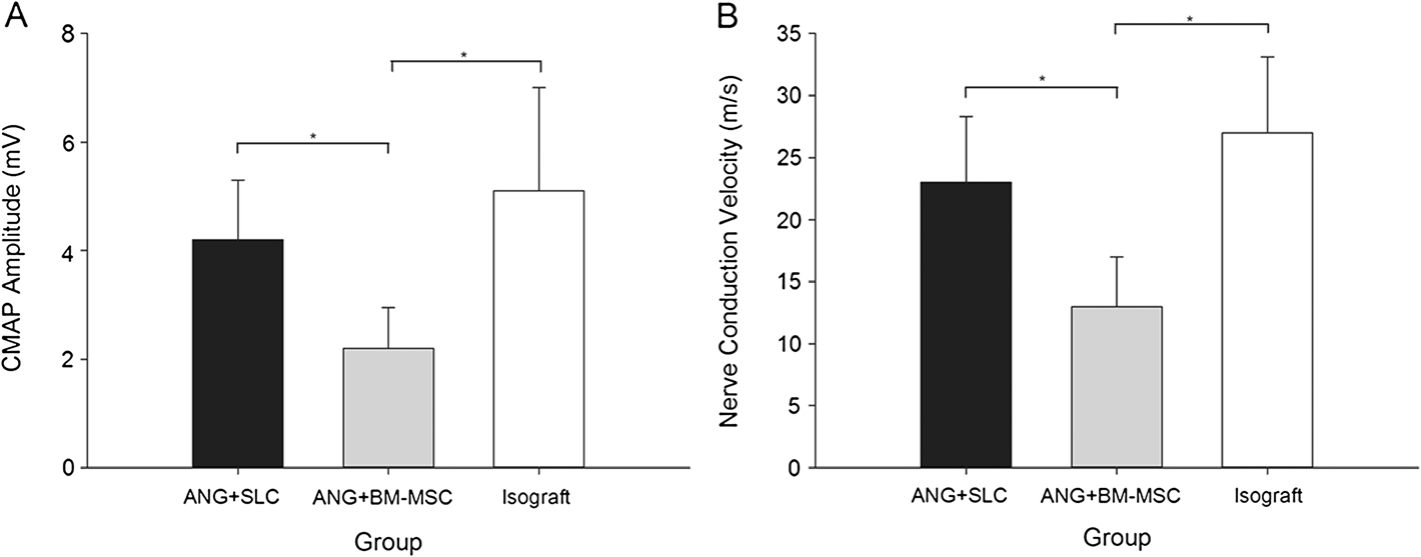
**Electrophysiological recovery analysis 12 weeks after grafting and experimental treatment.** The amplitude of the compound muscle action potential (CMAP) was significantly greater in the isograft and ANG + SLC groups than in the ANG + BM-MSC group **(A)**. The results of the nerve conduction velocity recording **(B)** were similar to those of the CMAP. The data are shown as the mean ± SEM, *P < 0.05 compared with the ANG + BM-MSC group (n = 15).

We also performed electrophysiological studies to determine whether the axons had regenerated through the entire length of the operated sciatic nerves. After 12 weeks, group C showed the best electrophysiological parameters (CMAP: 4.86 ± 1.81 mV; conduction velocity: 26.17 ± 5.11 m/s), followed by group A (CMAP: 4.21 ± 1.53 mV; conduction velocity: 22.64 ± 5.91 m/s) and group B (CMAP: 2.36 ± 1.03 mV; conduction velocity: 13.67 ± 4.98 m/s). Groups A and C did not differ in their electrophysiological tests (*P* > 0.05), but group C showed significantly subordinate results compared with the other groups (*P* < 0.05 for both tests, Figure 4).

**Figure 5:**
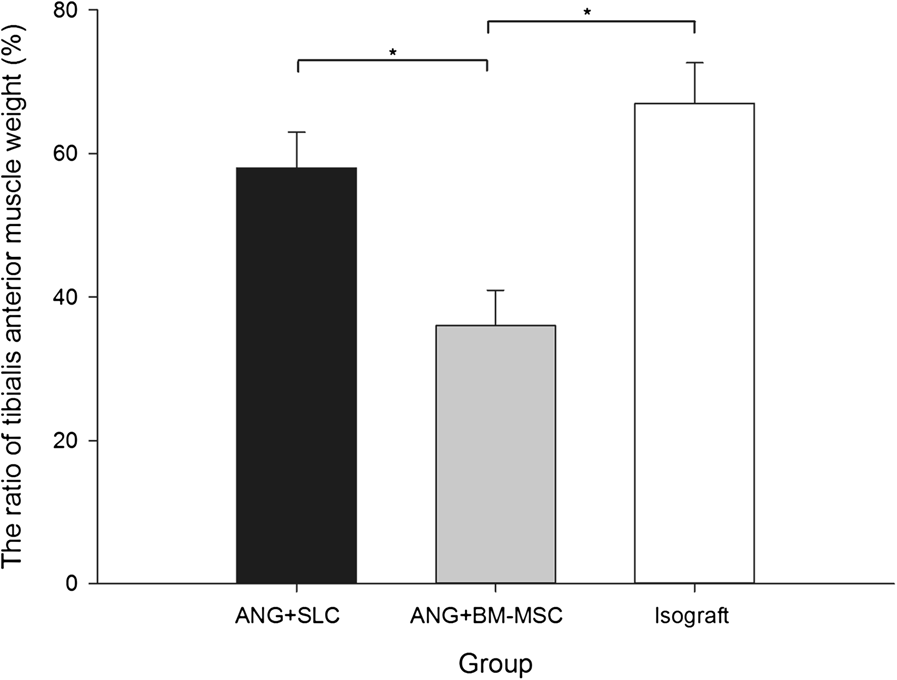
**The ratio of tibialis the anterior muscle weight.** The ratio of the tibialis anterior muscle weight in the ANG + BM-MSC group was significantly lower than that in the other two groups. The data are shown as the mean ± SEM, *P < 0.05 compared with the ANG + BM-MSC group (n = 15).

The results of the tibialis anterior muscle weight preservation of the different groups are shown in Figure 5. As an index of muscle atrophy, the tibialis anterior muscle weight ratio in group A (59.3 ± 4.6%) were significantly higher than in group B (38.5 ± 3.9%, *P* < 0.05, Figure 5). Moreover, no significant difference in the tibialis anterior muscle weight ratio was calculated between groups C (62.1 ± 4.5%) and A (*P* > 0.05, Figure 5), which indicates that acellular grafts with SCLs postpone muscle atrophy.

**Figure 6:**
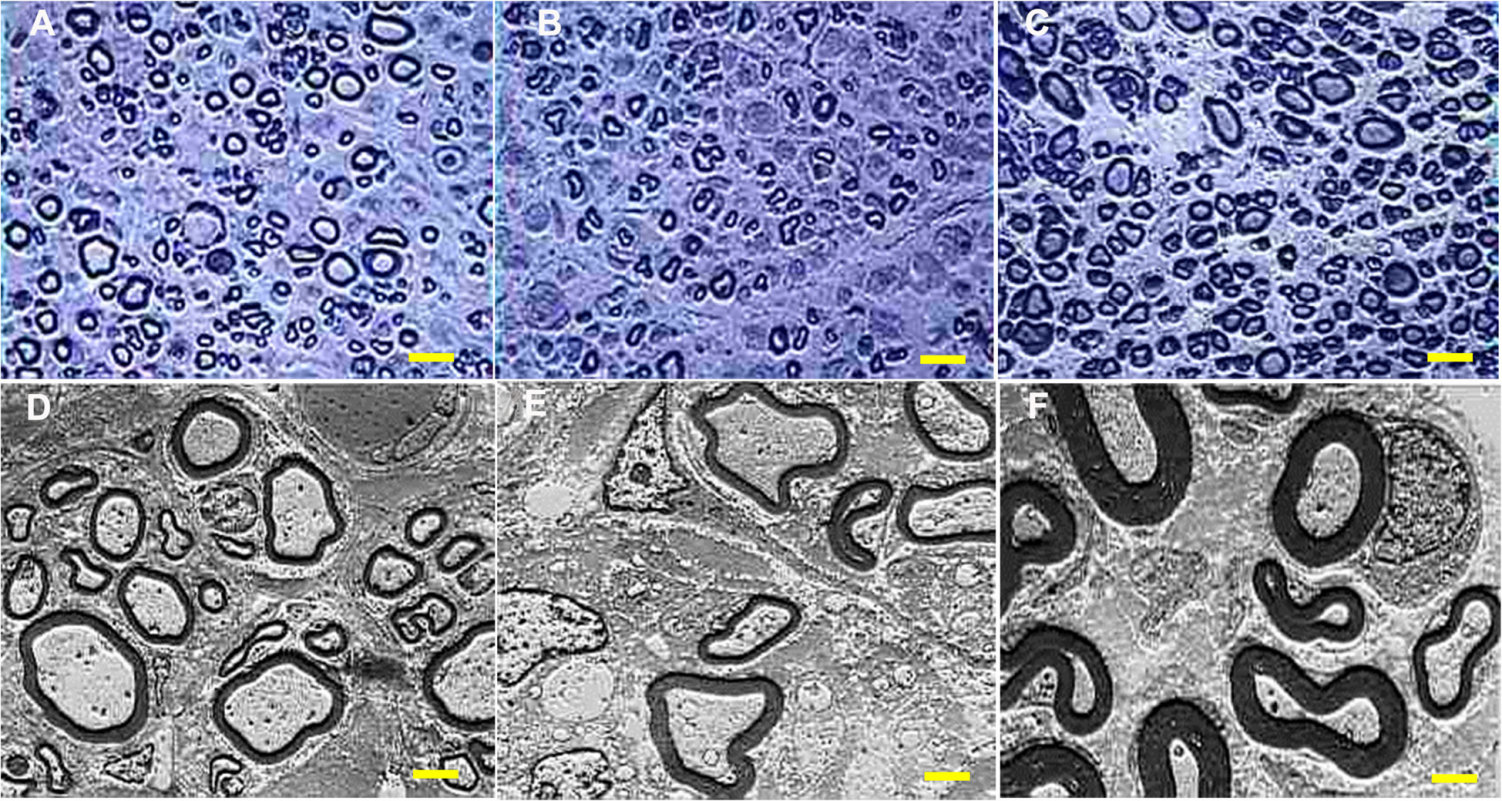
**Toluidine blue staining and transmission electron micrographs at 12 weeks postoperatively.** Toluidine blue staining (**A-C**, scale bar = 10 μm) and transmission electron micrographs (TEM, **D-F**, scale bar = 2 μm) at 12 weeks postoperatively in the ANG + SLC **(A and D)**, ANG + BM-MSC **(B and E)** and isograft **(C and F)** groups. The isograft group showed numerous large well-myelinated axons. The ANG + SLC group also demonstrated a large number of mature axons, whereas the ANG + BM-MSC group displayed generally fewer, small fibers.

**Figure 7:**
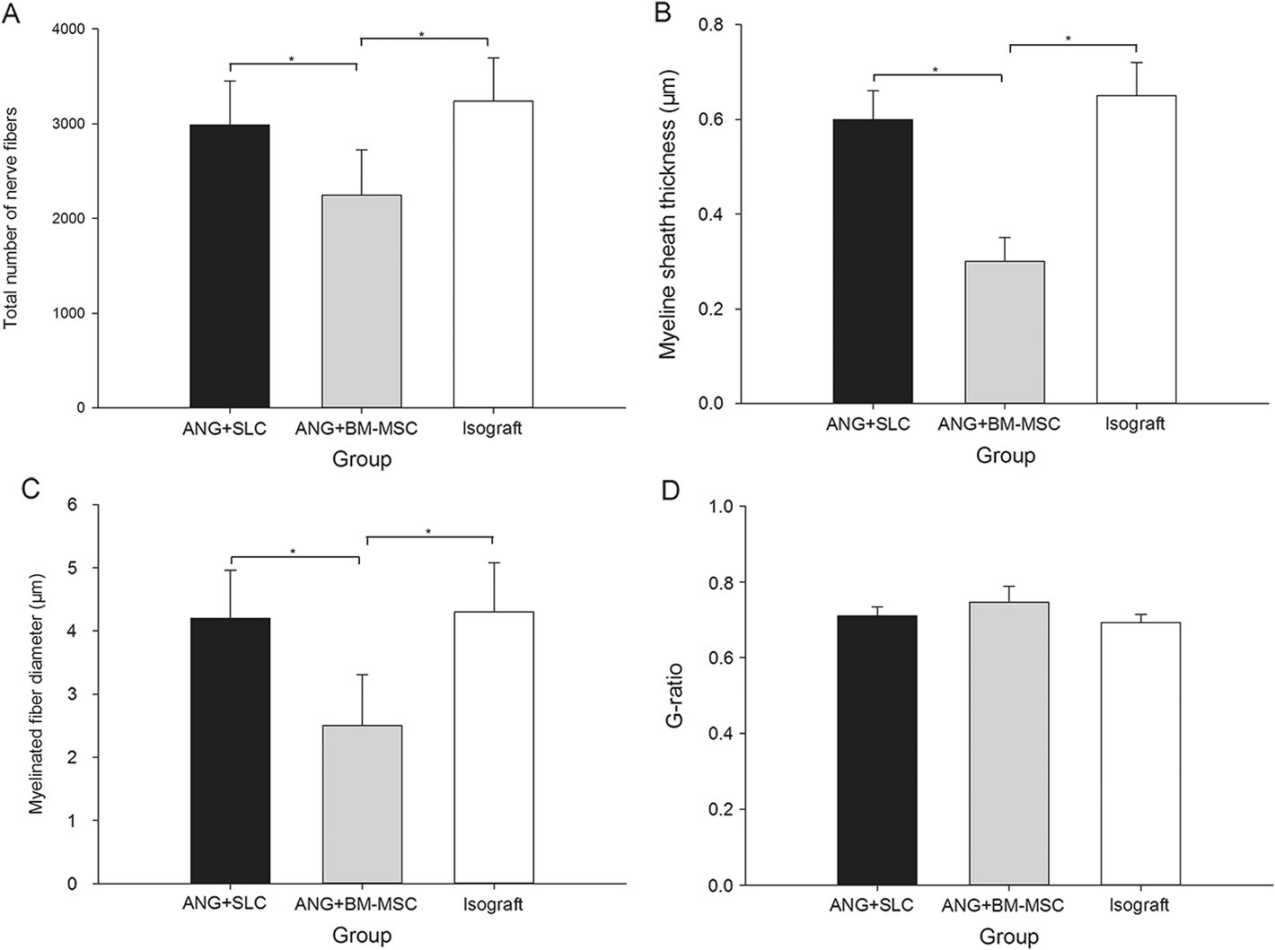
**Total number of nerve fibers, myelin sheath thickness, myelinated fiber diameter and G-ratio.** Total number of nerve fibers **(A)**, myelin sheath thickness **(B)**, myelinated fiber diameter **(C)** and G-ratio **(D)** at 12 weeks postoperatively. **A**, **B**, and **C** showed that the results from the isograft and ANG + SLC groups were significantly higher than those of the ANG + BM-MSC group. However, **D** showed no difference in the G-ratio among all groups. The data were shown as the mean ± SEM. *P <0.05, compared with the ANG + BM-MSC group (n = 15).

After repairing the 10-mm sciatic nerve gaps, histological examinations were performed. Toluidine blue staining and TEM were performed to assay whether the implanted nerve graft promoted axon regeneration. In Figures 6A-C and D-F, 12 weeks after transplantation, toluidine blue staining and TEM showed numerous myelin sheaths within the graft. However, group A showed superior results in the total number of nerve fibers (*P* < 0.05), myelin sheath thickness (*P* < 0.05) and myelinated fiber diameter (*P* < 0.05) compared with group B (Figure 7A-C). Additionally, groups A and C showed no differences in these three morphological indices (*P* > 0.05, Figure 7A-C). Furthermore, the G-ratios were not significantly different among the three groups (*P* > 0.05, Figure 7D).

**Figure 8:**
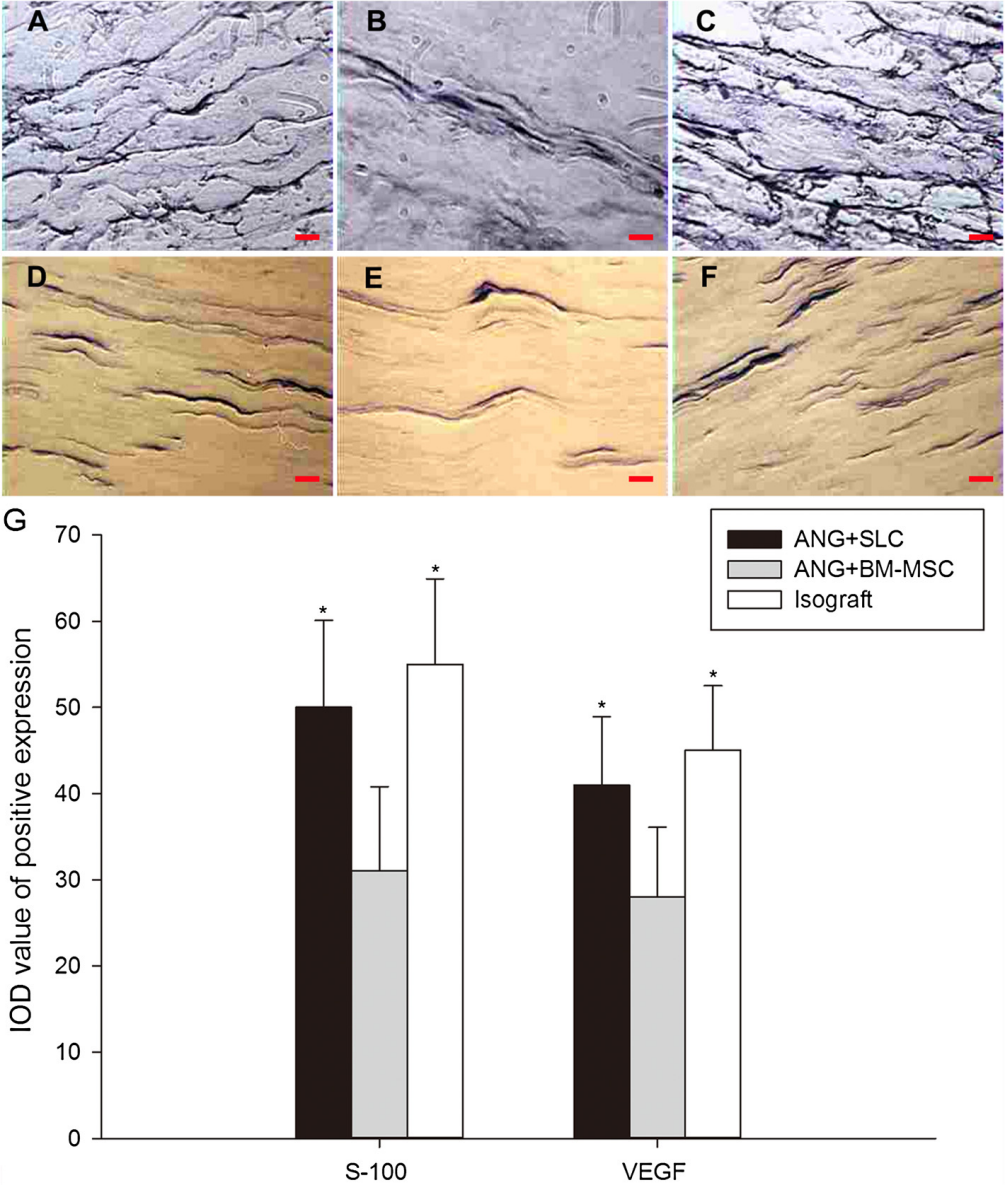
**S-100 and VEGF immunostaining and IOD value analyses at 12 weeks postoperatively.** S-100 immunostaining (**A-C**, scale bar = 10 μm) and VEGF immunostaining (**D-F**, scale bar = 20 μm) at 12 weeks postoperatively with SLCs **(A and D)**, BM-MSCs **(B and E)** and isografts **(C and F)**. The IOD values of the positive expression of S-100 and VEGF in the regenerated nerves **(G)**. The isograft and ANG + SLC groups showed a significantly higher level in both S-100 and VEGF immunostaining compared with the ANG + BM-MSC group. The data were shown as the mean ± SEM. *P <0.05, compared with the ANG + BM-MSC group (n = 15).

The figures of immunostaining revealed that compared with group B 12 weeks after surgery, group A showed a significantly higher level in both S-100 (*P* < 0.05) and VEGF (*P* < 0.05, Figure 8). At the same time, the S-100 and VEGF levels in groups A and C were similar (*P* > 0.05, Figure 8).

## Discussion

Peripheral nerve injury causes the loss of nerve functions, which results in the loss of neurological functional and an impaired quality of life [34,35]. Nerve regeneration and functional recovery after peripheral nerve injury is a clinical challenge. To overcome this problem, many studies and clinical trials have been performed. Autologous nerve bridges are the most effective way to induce axon regeneration and promote neurological recovery, but this technique is also restricted due to some limitations, such as a limited supply of nerve grafts [2]. The proper decellularization of peripheral nerves could reduce the immune response while retaining the internal structure and extracellular matrix components of the nerve [26,36]. However, with ANGs alone, the length of axon regeneration is limited [37]. The inclusion of neuronal support cells is critical for axonal proliferation [38]. Earlier studies revealed that the implantation of BM-MSCs within acellular nerve grafts can enhance the regeneration of sciatic nerve axons compared with ANGs [17,39-43]. However, some studies revealed that combining BM-MSCs with ANGs results in less axon regeneration compared with ANGs containing SCs [15,16,44]. Although the SC treatment induces good axon regeneration [27], it is rarely used due to their limited supply and the strong immune reactions they induce [13,14].

Based on previous studies, taking all the advantages together, we developed the idea of inducing BM-MSCs into Schwann cells, also known as Schwann-like-cells, and combining SLCs with ANG as the graft for peripheral nerve repair.

In this study, we created the acellular nerve through the use of trypsin, deionized distilled water and chemicals (Triton X-200, sulfobetaine-16 and sulfobetaine-10), the three key procedures, that were conducted based on the previously developed protocol [21]. This protocol removed cellular material from nerve tissue while preserving the natural ECM structure. Nerve grafts created with this decellularization protocol demonstrated the ability to support and guide regenerating nerves without eliciting the cell-mediated immune response typically associated with transplanted tissues, as shown in our study.

This study shows the strong plasticity of BM-MSCs by their differentiation into Schwann-like cells with the typical spindle-shaped Schwann cell morphology. Furthermore, SLCs and SCs have comparable functional properties, such as producing a number of neurotrophic factors and causing the neurite growth of neurons, which are not found in BM-MSCs [45]. Keilhoff et al. and Wang et al. demonstrated that SLCs can provide better nerve regeneration than BM-MSCs [16,20].

Here, we injected SLCs or BM-MSCs into the ANGs in equal cell suspension volumes at four evenly spaced locations [14,26]. Earlier histological evidence demonstrated that the injected cells will be well arranged along the longitudinal ANGs, although there will be damage of the basal lamina tubes caused by the microinjector [26,46]. Additionally, with this method, we could reduce the swelling of ANGs caused by suspension injection and the mechanical stress on injected cells and obtain better cell survivals after injection [27].

We selected SFI as a parameter to evaluate the functional recovery after nerve repair [47]. As mentioned previously, a SFI value of approximately 0 indicates normal nerve function and a value of approximately −100 indicates total dysfunction. The SFI of the nerve repair group using acellular nerve grafts supplemented with SLCs was significantly greater than that of the nerve repairs with the acellular nerve grafts combined with BM-MSCs (*P* < 0.05). In addition, the functional recovery of the SLC group was similar to that of the nerve repair with the isograft (*P* > 0.05).

Studies demonstrated that foetal neural stem cells transplanted into peripheral nerves after injury can improve the formation of functional neuromuscular junctions with denervated muscle by inducing axon regeneration, which reduces muscle atrophy after peripheral nerve injury [48]. Based on this theory, we chose the tibialis anterior muscle weight to evaluate nerve regeneration. We found that the acellular nerve grafts supplemented with SLCs used for nerve grafts can better maintain muscle weight compared with nerve repairs with the acellular nerve grafts combined with BM-MSCs (*P* < 0.05). Furthermore, the SLC group showed equivalent results to those of the isograft group in muscle weight (*P* > 0.05). In this study, we also chose the nerve electrophysiological function test for evaluating nerve recovery. As previously known, better nerve regeneration and axonal myelination would result in higher amplitude and nerve velocity. We found that the amplitude of the compound muscle action potential and nerve conduction velocity from the SLC nerve repair group showed significantly higher results in electrophysiological function (*P* < 0.05) compared with the BM-MSC group and that the SLC group presented almost equal CMAP and nerve conduction velocity data compared with the isograft group in the nerve electrophysiological function test (*P* > 0.05).

Although the number of nerve fibers, myelinated fiber diameter, and myelin sheath thickness in the ANG containing SLCs group were similar to those in the isograft group (*P* > 0.05), the ANGs containing SLCs produced a significantly higher number of nerve fibers, greater myelinated fiber diameter and greater myelin sheath thickness than the BM-MSC group (*P* < 0.05), which indicated that SLCs were beneficial for improving the nerve regeneration and maturation of myelinated fibers compared with the BM-MSC group. The G-ratio (ratio of axon diameter to fiber diameter) is widely used as a functional and structural index of optimal axonal myelination [49]. An increase in the G-ratio of fibers may suggest a decrease in myelination, which has a bad effect on nerve repair. Although we found no significant difference among all groups (*P* > 0.05), we could still show a trend in nerve myelination in the three groups, as we determined at an early time point in the recovery.

The demonstrated better potency of axon generation in the SLC group compared with the BM-MSC group is reflected in the expression patterns of the calcium-binding protein S-100 (Figure 8G). After being released into the extracellular space, S-100 stimulates proliferation, neuronal survival, and differentiation [50]. Therefore, the differences in supporting nerve regeneration may easily be attributed to the different levels of this neurotrophin. Rong et al. [51], demonstrated that blocking S-100 receptors suppresses peripheral nerve regeneration.

VEGF is a peptide associated with angiogenesis. It can promote endothelial cell division and enhance neovascularization. Additionally, VEGF directly influences the behaviour of nerve cells by the neurotrophic and neuroprotective influences [52,53]. Furthermore, the VEGF treatment of acellular peripheral nerve isografts enhances axon sprouting, resulting in an increased number of regenerating axons [54]. Studies also noted that VEGF stimulates Schwann cell invasion and the neovascularization of acellular nerve grafts [52]. Based on these studies, we chose VEGF as an indicator of axon regeneration. We found that the SLC group showed a greater expression of VEGF (significantly higher IOD value, *P* < 0.05) than the BM-MSC group and that the SLC group showed an equivalent IOD value compared with the isograft group (*P* > 0.05).

## Conclusions

In conclusion, this study demonstrated that the combination of an acellular nerve graft with SLCs provides a microenvironment that increases axon elongation and myelinization. Meanwhile, the nerve regeneration is facilitated when compared with the combination of an acellular nerve graft and BM-MSCs in rats. Additionally, considering the disadvantages of isografts, supplementing SLCs around the acellular nerve graft might be a better candidate for the treatment of a peripheral nerve defect. Further investigation is needed to study the mechanism and determine the conditions to optimize this technique to improve nerve regeneration when used for peripheral nerve repair.

## Abbreviations

SLC: Schwann-like cell; BM-MSC: Bone marrow-derived mesenchymal stem cell; ANG: Acellular nerve graft; VEGF: Vascular endothelial growth factor; ECM: Extracellular matrix; SC: Schwann cell; SD: Sprague–Dawley; SB-10: Sulfobetaine-10; SB-16: Sulfobetaine-16; DMEM: Dulbecco’s modified Eagle’s medium; αMEM: Minimum Essential Medium; BME: Beta mercaptoethanol; FBS: Fetal calf serum; at-RA: All-trans-retinoic acid; FSK: Forskolin; bFGF: Basic fibroblast growth factor; PDGF: Platelet-derived growth factor; HRG: Heregulin; SFI: Sciatic function index; PL: Print length; TS: Toe spread; ITS: Intermediary toe spread; CMAP: Compound muscle action potential; IOD: Integrated optical density; SEM: Standard error of the mean; ANOVA: One-way analysis of variance; TEM: Transmission electron micrograph; Lihong Fan: Zefeng Yu and Jia Li equally contributed to this work.

## Competing interests

The authors declare that they have no financial or non-financial competing interests.

## Authors' contributions

LF and ZY carried out the experiments and drafted the manuscript. JL performed the statistical analysis and drafted the manuscript. XD did literature review and helped to carry out the experiments. KW participated in the design and coordination of the study, and helped to draft the manuscript. All authors read and approved the final manuscript.

## Pre-publication history

The pre-publication history for this paper can be accessed here:

http://www.biomedcentral.com/1471-2474/15/165/prepub
